# Isolation, Molecular Characterization, and Antibiotic Resistance of Avian Pathogenic *Escherichia coli* in Eastern China

**DOI:** 10.3390/vetsci9070319

**Published:** 2022-06-25

**Authors:** Dossêh Jean Apôtre Afayibo, Hong Zhu, Beibei Zhang, Lan Yao, Hosny Ahmed Abdelgawad, Mingxing Tian, Jingjing Qi, Yali Liu, Shaohui Wang

**Affiliations:** Shanghai Veterinary Research Institute, Chinese Academy of Agricultural Sciences, Shanghai 200241, China; jeanafayibo@gmail.com (D.J.A.A.); zhuhong0526152x@126.com (H.Z.); zhang05202022@126.com (B.Z.); yaolan0611@126.com (L.Y.); hosnyahmed@vet.aswu.edu.eg (H.A.A.); tianmx530@126.com (M.T.); qijingjing@shvri.ac.cn (J.Q.); liuyali@shvri.ac.cn (Y.L.)

**Keywords:** avian pathogenic *Escherichia coli* (APEC), serotypes, phylogenetic group, virulence genes, antibiotic resistance

## Abstract

Avian pathogenic *Escherichia coli* (APEC) causes colibacillosis in avians, resulting in considerable losses in the poultry industry. APEC showed zoonotic potential initially related to the fact that APEC serves as the reservoir of virulence genes and antibiotic resistance genes for other *E. coli*. Thus, we determine the serotypes, phylogenetic groups, virulence genes distribution, and antibiotic resistance profiles of APEC isolates in eastern China. A total of 230 APEC were isolated from diseased chicken and duck with typical colibacillosis symptoms. Serotyping identified that O78 (44.78%) was the predominant serotype. The majority of APEC isolates were classified into B2 (29.57%), A (26.96%), D (20.00%), and B1 (18.26%), respectively. Among the 15 virulence genes, a high prevalence of *ibeB* (99.57%), *fimC* (91.74%), *mat* (91.30%), *ompA* (83.04%), and *iss* (80.43%) genes was observed. Except for low resistance rates for imipenem (1.7%) and polymyxin B (0.4%), most of the APEC isolates were resistant to erythromycin (98.7%), enrofloxacin (96.1%), tetracycline (95.2%), doxycycline (93.9%), lincomycin (90.0%), and streptomycin (90.0%). Moreover, all APEC exhibit multi-drug resistance. This study indicated that APEC isolates harbor a variety of virulence genes and showed multi-antibiotic resistance profiles, providing proof for understanding the epidemiological background and zoonotic potential of APEC in poultry farms.

## 1. Introduction

Extraintestinal pathogenic *E. coli* (ExPEC) are facultative pathogens that constitute a portion of fecal flora in most healthy humans, other mammals, and birds. Several groups, including uropathogenic *E. coli* (UPEC), neonatal meningitis *E. coli* (NMEC), sepsis-associated *E. coli* (SEPEC), and avian pathogenic *E. coli* (APEC), belong to ExPEC [1]. Certain APECs could cause pericarditis, air sacculitis, perihepatitis, peritonitis, and other primarily extraintestinal infections collectively referred to as colibacillosis [2]. The distribution of APEC virulence traits is related to diverse geographic areas. Several studies have shown that APEC may cause colibacillosis by enhancing the pathogenicity of virulence factors encoded by diverse genes, including *cva/cvi*, *iroN*, *iss*, *iutA*, *sitA*, *tsh*, *fyuA*, *irp2*, *ompT*, and *hlyF* [3,4]. Nevertheless, the pathogenic mechanism of APEC is still unidentified. Moreover, the control and prevention measurements of avian colibacillosis in poultry are limited. Thus, it is necessary to dissect the pathogenic mechanism of APEC by identifying the target virulence genes and multidrug-resistance which could be supportive for new potential drugs to control APEC.

Distinguishing APEC isolates from other pathogenic *E. coli* strains requires identifying diverse serotypes and phylogenic groups. When compared to other extraintestinal pathogenic *E. coli* (ExPEC), the majority of APEC isolates might be O1, O2, O18, and O78 serotypes [5]. To classify *E. coli* strains into the A, B1, B2, and D phylogroups, in a previous study, a phylotyping method was developed using the following three specific genes: *yjaA*, *chuA*, and *TspE.4C2* [6]. Pathogenic strains may belong to B2 and D groups, whereas commensal strains fall into A and B1 groups [7]. B1 and C phylogroups were mostly identified among APEC from yolk sac infection (YSI) and septicemia isolates [7]. The ExPEC, including APEC strains, were grouped under a highly prevalent phylogroup B2 and lower prevalent group D [8,9].

The primary method of controlling APEC infections in poultry is to use a variety of antibiotics [10]. Meanwhile, the development of multidrug-resistant strains of APEC is a severe problem for global public health, with a significant impact on animal health and food safety [11]. The emergence of antibiotic-resistant *E. coli* strains has become a global issue due to the overuse of antibiotics in the poultry industry [12]. The antibiotic-resistant forms of APEC strains were found to activate antibiotic resistance genes in other pathogenic *E. coli* strains, and such resistance genes could easily be transmitted and spread between animals and humans [13].

Here, we determined the prevalence of serotypes, phylogenetic groups, virulence genes, and antibiotic resistance profiles of APEC isolates. Our findings may contribute to understanding the epidemiological factors and zoonotic potential of APEC in poultry.

## 2. Materials and Methods

### 2.1. Sample Collection, Isolation, and Identification of APEC

All the samples were collected from diseased chicken (*n* = 137) and duck (*n* = 93) with colibacillosis from farms in Eastern China (Jiangsu, Anhui, Fujian, and Shandong provinces). All birds showed typical clinical symptoms and pathological lesions of colibacillosis, such as perihepatitis, pericarditis, air sacculitis, omphalitis, and peritonitis. The liver, spleen, and lung were collected aseptically for bacteria isolation. The samples were subjected to MacConkey agar plate and cultured overnight at 37 °C. The suspected bacterial single clones were selected and grown in Luria Bertani (LB) broth for further identification. Extraction of the bacterial DNA was carried out by boiling and rapid cooling methods as previously described [14]. Briefly, *E*. *coli* was collected and resuspended in 100 μL of nuclease-free water and boiled for 10 min. The supernatant was collected and used as the DNA template for *E. coli* identification and virulence genes detection [14]. The bacteria isolates were examined and identified by PCR targeting the *E. coli* alkaline phosphatase *phoA* gene. All *E. coli* isolates were cultured in LB at 37 °C and stored in 50% glycerol at −80 °C until further characterization.

### 2.2. Serotyping

PCR and the traditional serum agglutination methods were used to determine the O serotypes of all the samples as described previously [14]. The serotyping was performed by targeting primers designed for the common APEC predominant serotypes [15,16]. A traditional serum agglutination test was conducted with the specific serum against *E. coli* O antigens (Statens Serum Institute, Copenhagen, Denmark) following the manufacturer’s recommendations.

### 2.3. Identification of Phylogenic Groups

The phylogenetic groups of APEC isolates were investigated using triplex PCR targeting *chuA, yiaA,* and *TspE4.C2*, as previously reported by Clermont and coworkers [17]. The APEC strains were classified according to the PCR results.

### 2.4. Detection of Virulence Genes

The 230 APEC isolates were tested for the presence of 15 virulence genes by multiplex PCR and simplex PCR simultaneously, as described previously [15]. Multiplex PCR assays were designed to detect the following simultaneously: (1) *ompA* (919 bp), *neuC* (676 bp), *fimC* (497 bp); (2) *ibeA* (342 bp); (3) *cva/cvi* (598 bp); (4) *vat* (981 bp), *mat* (899 bp), *fyuA* (774 bp), *irp2* (288 bp); (5) *tsh* (805 bp), *iucD* (693 bp), *papC* (483 bp), *iss* (309 bp); (6) *ibeB* (1172 bp) and *iroN* (866 bp), which were identified. The primers used for virulence genes detection were described previously [5,18,19,20,21,22,23] (Supplementary Table S1). All primers were synthesized commercially (Sangon Inc., Shanghai, China). PCR procedures were performed in a 25 μL reaction mixture, including 12.5 μL 2×Rapid Taq Master Mix (Vazyme Biotech Co., Ltd., Nanjing, China), 0.5 μL of the forward and reverse primers (100 μM), and 1 μL template DNA, supplemented with appropriate volumes of sterile ddH_2_O. Sterile distilled water was used as a negative control while APEC O1 and DE719 [15,24] were used as positive controls in PCR assays. PCR reactions were carried out under the following conditions: pre-denaturation at 95 °C for 5 min, followed by 30 cycles of 95 °C for 35 s, 57 °C for 30 s, 72 °C for 40–60 s, and a final extension at 72 °C for 10 min [25]. The PCR amplicons were analyzed by agarose gel electrophoresis with 1% agarose gel and photographed at UV exposure. The PCR products size was determined and compared to the DNA Marker (Takara, Dalian, China).

### 2.5. Antibiotic Susceptibility Testing

Each APEC isolate was tested for antibiotic susceptibility using the Kirby–Bauer disk diffusion method [26]. In brief, a total of 24 antibiotics were selected for testing, including amoxicillin (AMX), ampicillin (AMP), imipenem (IPM), ceftriaxone (CRO), lomefloxacin (LOM), cefalotin (CF), ceftazidime (CAZ), spectinomycin (SPT), ciprofloxacin (CIP), doxycycline (DOX), tetracycline (TET), chloramphenicol (CHL), lincomycin (L), kanamycin (KAN), streptomycin (STR), erythromycin (ERY), florfenicol (FFC), cefotaxime (CTX), polymyxin B (PMB), enrofloxacin (ENR), trimethoprim (TMP), gentamicin (GEN), sulfamethoxazole (SXT) and amikacin (AMK). These antibiotics belong to different classes of drugs and were chosen according to commonly used in clinical practice as mentioned in the previously study [27]. After APEC being cultured onto ordinary agar plates, the susceptibility paper disks impregnated with the 24 antibiotics were affixed to the surface of the agar medium. The plates were incubated overnight at 37 °C, and the diameters of the inhibition area were recorded. The antibiotic susceptibility results were observed and described following the manual of the Clinical and Laboratory Standards Institute (CSLI) as follows: susceptible (S), intermediately resistant (I), or resistant (R) [28].

### 2.6. Statistical Analysis

Data analysis was performed using the GraphPad Software manual (GraphPad Software, La Jolla, CA, USA) and microsoft Excel 2016.

## 3. Results

### 3.1. Isolation and Identification of APEC Isolates

The diseased, dead chickens and ducks with typical colibacillosis symptoms or pathological lesions were collected for bacteria isolation with selective medium culture and PCR identification. In total, 230 *E. coli* strains were isolated from the extraintestinal tissues of birds. Among them, 137 strains were identified from chickens, and 93 from ducks.

### 3.2. Serotype Identification

**Figure 1:**
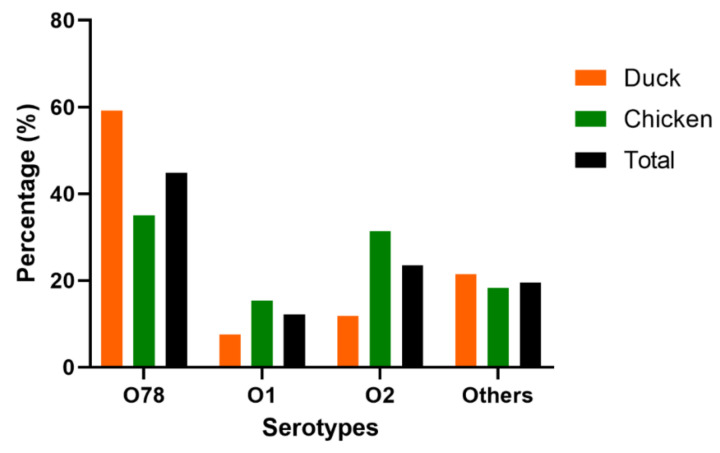
The serotypes of 230 APEC isolates from chickens (*n* = 137) and ducks (*n* = 93). Bar charts show the serotypes distribution of APEC isolated from chicken (green), duck (orange) and total (black).

PCR and serum agglutination tests showed that approximately 80% of the 230 APEC isolates belong to O1 (12.17%, 28/230), O2 (23.48%, 54/230), and O78 (44.78%, 103/230) serotypes. Other serotypes, such as O8, O9, and O18, accounted for approximately 19.57% (45/230). These findings revealed that O78 is the predominant serotype of the APEC isolates. Although the proportions of the serotypes in the two birds were different, O1, O2, and O78 exhibited similar trends. In ducks and chickens, the dominant serotype was O78 (59.14 and 35.04%, respectively), followed by O2 (11.83 and 35.39%, respectively) and O1 (7.53 and 15.33%, respectively), indicating O78 as the most virulent APEC in Eastern China (Figure 1 and Table S2).

### 3.3. Phylogenetic Grouping

**Figure 2:**
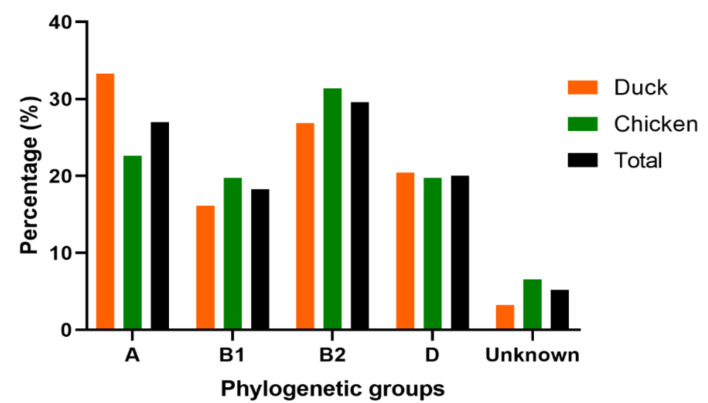
Phylogenetic classification of 230 APEC isolates from chickens (*n* = 137) and ducks (*n* = 93). Bar charts show the phylogenetic groups distribution of APEC isolated from chicken (green), duck (orange) and total (black).

The phylogenetic analysis classified the APEC isolates mostly into B2 (29.57%, 68/230), A (26.96%, 62/230), D (20.00%, 46/230), and B1 (18.26%, 42/230), sub-groups (Figure 2 and Table S3). However, twelve (12) APEC isolates (5.22%) were not assigned to any group. The dominant phylogenetic group in chicken was B2 (31.39%), followed by A (22.63%), and B1 and D (19.71%, respectively). Meanwhile, in ducks, the predominant phylogenetic group was A (33.33%), followed by B2 (26.88%), D (20.43%), and B1 (16.13%), indicating that the two birds have different levels of prevalence (Figure 2 and Table S3).

### 3.4. Distribution of Virulence Genes

**Figure 3:**
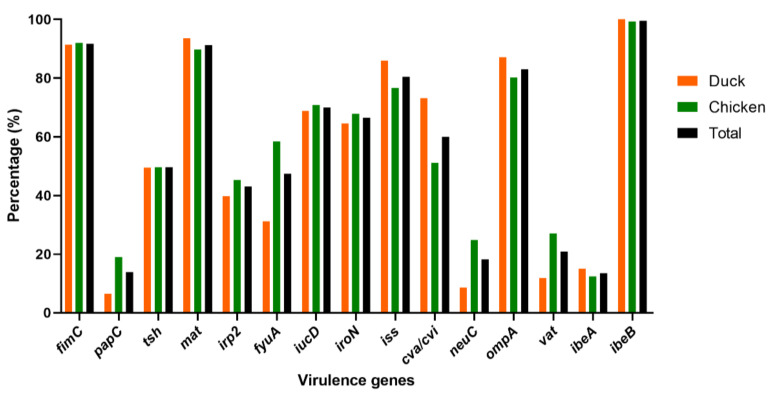
Distribution of virulence genes in 230 APEC isolates from chickens (*n* = 137) and ducks (*n* = 93). Bar charts show the frequency rates of each virulence gene in APEC isolated from chicken (green), duck (orange) and total (black).

The virulence genes distribution encoding to adhesins, iron acquisition systems, protectins, toxins, and invasins, were determined in this study. According the PCR detection, the highest detection rates were found for genes *ibeB* (229/230, 99.57%), *fimC* (211/230, 91.74%), *mat* (210/230, 91.30%), *ompA* (191/230, 83.04%), and *iss* (185/230, 80.43%) (Figure 3 and Table S4). The *tsh*, *irp2*, *fyuA*, *iroN*, *iucD*, and *cva*/*cvi* genes were prevalent in 40–81% of 230 APEC isolates. Furthermore, low prevalence rates were observed in genes *vat* (48/230, 20.87%), *neuC* (42/230, 18.26%), *papC* (32/230, 13.91%), and *ibeA* (31/230, 13.48%). All APEC strains in this study harbored at least three (3) virulence genes.

To identify the most virulent genes in the two birds for future genomic studies, we compared their distribution. The results showed that the dominant virulence genes in both chickens and ducks were *fimC*, *mat*, *iss*, *ompA*, and *ibeB* with approximatively the same percentages (Figure 3 and Table S4). Of the other virulence genes, *papC*, *irp2*, *fyuA*, *neuC*, and vat showed high distribution in chickens (18.98, 45.26, 58.39, 24.82, and 27.01%, respectively) compared to ducks (6.45, 39.78, 31.18, 8.60, and 11.83%, respectively). The *tsh*, *iucD*, *iroN*, and *ibeA* genes exhibited similar rates in chickens (49.64, 70.80, 67.88, and 12.41%, respectively) and ducks (49.46, 68.82, 64.52, and 15.05%, respectively) (Figure 3 and Table S4).

### 3.5. Association of Virulence Genes Distribution to Serotype, Phylogenetic Group

**Figure 4:**
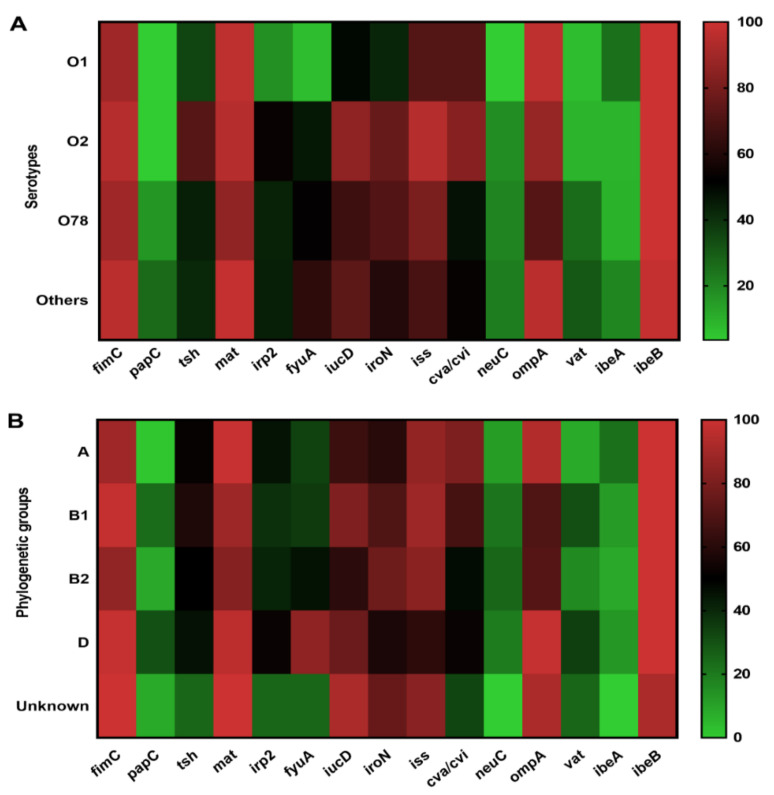
The prevalence of the virulence genes distribution to serotypes and phylogenetic groups (negative, weak, moderate, and strong) among APEC isolates. (**A**) Prevalence of the virulence genes distribution and the serotypes. (**B**) Prevalence of the virulence genes distribution and the serotypes and the phylogenetic groups.

The correlation between the virulence genes distribution and O serotype was examined. Most virulence genes among serotypes O1, O2, and O78 showed similar patterns and some serotypes showed positive correlations with some virulence genes (Figure 4A and Table S5). The genes *ibeB*, *fimC*, *mat*, *iss*, and *ompA* were widely detected in O1, O2, and O78 serotypes. Genes *irp2*, *fyuA*, *iroN*, and *neuC* were predominant in O2 and O78 serotypes compared to O1 APEC isolates. The *papC* and vat genes showed low prevalence in O1 and O2 serotypes compared to O78 and other serotypes. Gene *ibeA* was mainly present in O1 (25%) and other serotypes (20%), respectively (Figure 4A and Table S5).

The relationship between virulence genes and phylogenetic groups is shown in Figure 4B and Table S6. We found that *fimC*, *tsh*, *mat*, *irp2*, *iroN*, *iss* and *ibeB* genes were widely distributed in all groups. Genes *papC* and *vat* were mainly detected in groups B1 and D compared to groups A and B2. Genes *fyuA*, *iucD*, and *cva*/*cvi* were extensively detected in groups D (84.78%), B1 (80.95%), and A (80.65%), respectively. In addition, *neuC* and *vat* genes were less present in group A strains, whereas A was the predominant group that harbored the gene *ibeA* (Figure 4B and Table S6).

### 3.6. Antibiotic Susceptibility Testing

**Figure 5:**
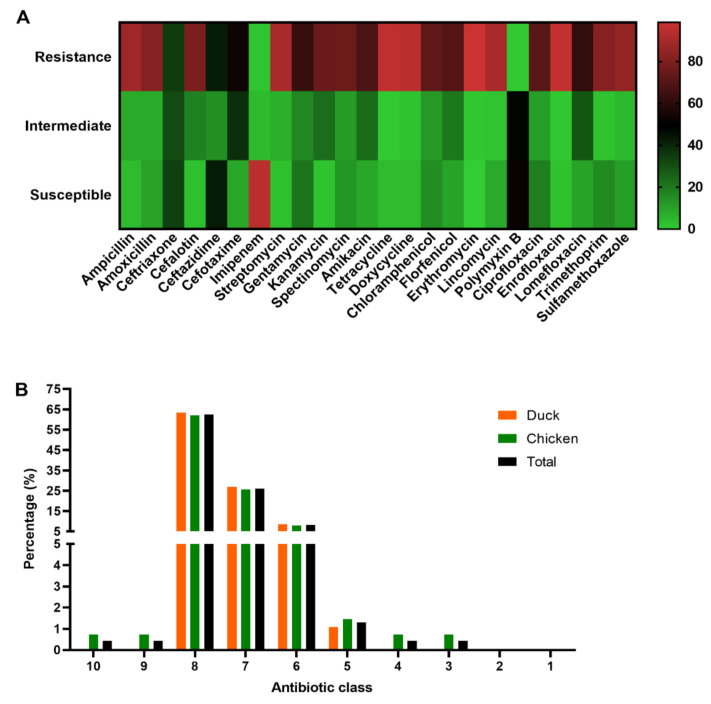
Antibiotic susceptibility testing. (**A**) Information of the antibiotic susceptibility testing (negative, weak, moderate, and strong) profiles of the 230 APEC isolates. (**B**) Prevalence of the MDR of 230 APEC isolates from chickens (*n* = 137) and ducks (*n* = 93). Bar charts show the frequency rates of MDR from chicken (green), duck (orange) and total (black).

The antibiotic susceptibility testing results of the 230 APEC isolates revealed that they were highly resistant to erythromycin (98.7%), enrofloxacin (96.1%), tetracycline (95.2%), doxycycline (93.9%), lincomycin (90.0%), streptomycin (90.0%), ampicillin (87.8%), sulfamethoxazole (84.3%), amoxicillin (81.7%) and cefalotin (78.7%). APEC isolates showed relative resistance to florfenicol (69.6%), amikacin (67.4%), gentamicin (62.2%), lomefloxacin (61.3%), and cefotaxime (52.6%). However, we observed a low resistance rate to imipenem (1.7%) and polymyxin B (0.4%) (Figure 5A and Table S7). It was worth noting that both the 230 APEC isolates were resistant to at least three (3) different categories of antibiotics, indicating that they are multi-drug resistant (MDR) strains [29]. The number of APEC isolates that showed resistance to 8, 7, and 6 antibiotic classes was 144 (62.61%), 60 (26.09%), and 19 (8.26%), respectively. The proportion of isolates resistant to 10, 9, 4 and 3 antibiotic categories was 1 (0.43%) each (Figure 5B and Table S8).

## 4. Discussion

Animal poultry has grown expeditiously in several areas of China over the last few decades [30]. Many studies have demonstrated the impact of colibacillosis, one of the major diseases that cause death and economic loss in the poultry industry [31]. Antibiotics are used as a control measure in these situations. Unfortunately, the use of antibiotics to treat APEC contaminations occasionally results in the emergence of multidrug-resistant APEC strains [32]. Even though many studies have been conducted in China, information and data on virulence genes and antibiotic resistance in APEC are scarce. The current study determined virulence genes and antibiotic resistance in infected chickens and ducks.

The distribution of APEC isolates was determined during further glass plates agglutination, tube agglutination to specific O antigens and PCR testing. Only O1, O2, and O78 were found to be associated with virulence genes in this study. These serotypes were found at a prevalence of 12.17%, 23.48%, and 44.78%, respectively, with O78 predominance. This result is consistent with previous findings in Egypt [33]. The widespread distribution of these serotypes among APEC strains may attest to their importance in extraintestinal infections. Furthermore, O78 was shown to have zoonotic potential with severe human diseases [34]. According to the previous sequencing study, O78 and O1 serotypes might induce genetic diversity in one another, and diverse core-genome types might be modified to generate the same avian disease through distinct mechanisms [35]. In the present study, the predominant serotype O78 showed 35.04% prevalence in chickens and 59.14% in ducks. This result is similar to the previous study conducted in South Korea [36], which reported that O78 serotype was predominant in duck isolates (88.9%) compared to chicken isolates (29.9%).

The epidemiological distribution of APEC strains in different parts of the world led to their classification into phylogroups A and D [37]. In the previous study conducted by Clermont and coworkers, *E. coli* strains were classified into A, B1, B2, and D groups whereas the virulent extra-intestinal pathogenic *E. coli* (ExPEC) strains belonged mostly to B2 and D phylogenetic groups [17]. Our current study revealed a strong predominance of B2 (29.57%), followed by A (26.96%), B1 (18.26%), and D (20.0%) groups. In view of the significant rate of APEC, B2 (29.57%) and A (26.96%) phylogenetic groups detected in our current study, and similar to earlier studies [30,38], we inferred that poultry samples could be a potential reservoir of APEC. Another study on the phylogenetics of APEC isolates in China showed diversity and reported that most APEC isolates fell into group A followed by D, B1, and B2 [11,39]. The phylogenic group B2 is most commonly associated with APEC primary infection. Furthermore, it is thought to have an important amount of virulence genes and to be more virulent during ExPEC infections [40].

Virulence genes play important roles and are commonly associated with pathogenicity in APEC during infections [41]. Adhesins, iron acquisition systems, protectins, toxins, invasins, metabolism, and secretion systems were the most common virulence genes [41,42]. In the current study, *fimC*, *mat*, *ompA*, *iss*, *iucD*, *iroN*, *cva/cvi*, *tsh*, *fyuA*, *irp2*, *vat*, *neuC*, *papC*, and *ibeA, ibeB*, genes were identified. The majority of the virulence genes were detected with a frequency of greater than 50%, confirming the high genetic variability of APEC isolates. *ibeB* predominated with a frequency of 99.57%, which corresponded to over 97% of the sequence homology observed by Wang and colleagues [19]. It may contribute to the invasion of the brain microvascular endothelial cells (BMEC) and resistance to oxidative stress, biofilm formation, colonization, and proliferation [19,43].

APEC isolates contained a high frequency of virulence genes such as *fimC*, *mat*, *ompA*, *iss*, *iucD*, *iroN*, and *cva/cvi* (91.74%, 91.30%, 83.04%, 80.43%, 70.0%, 66.52%, and 60.0%, respectively). The frequency of virulence genes detected in this study is comparable to other studies conducted in Portugal and China, which revealed high prevalence rates among APEC strains [44,45]. Furthermore, previously conducted research in eastern China confirmed the high prevalence of *fimC* and *ompA* [17]. Several genes encoding these fimbriae, as well as the adhesins *fimC* and *mat*, may aid in adherence to other cell surfaces during the early stages of APEC infections [1]. The *ompA* gene was found to be involved in the synthesis of a bacterial outer membrane protein [46]. The virulence genes *iss*, *tsh*, *iroN*, and *cva/cvi* may play roles in the pathogenesis of APEC infections [1,47]. In this study, the 15 tested virulence genes showed almost similar patterns in chicken and duck isolates. On the contrary, Jiyeon Jeong and coworkers reported that chicken isolates may have a higher virulence potential than duck isolates [48]. Our findings suggest that chicken and duck isolates may possess a similar potential for virulence genes. Moreover, they indicate the need for sequencing *fimC, mat, ompA, iss, iucD, iroN,* and *cva/cvi* genes and performing comparative genomic studies for the efficient control of APEC diseases.

Antibiotics were widely used as control measurements in many farms in east China due to APEC infections. This method was found to be ineffective due to APEC strains’ multidrug resistance. Herein, we found that at least one APEC isolate was resistant to all tested antibiotics. The isolates were highly resistant to erythromycin, enrofloxacin, tetracycline, doxycycline, lincomycin, streptomycin, ampicillin, sulfamethoxazole, and amoxicillin. This result is consistent with previous reports from China [11]. Furthermore, the present findings also agree with earlier reports that showed high resistance to ampicillin, amoxicillin, streptomycin, and tetracycline [49]. It could be due to the overuse of antibiotics in treating APEC contaminations in eastern China. Resistance to antibiotics such as ampicillin, tetracycline, erythromycin, sulfamethoxazole, and doxycycline streptomycin is the most common according to a report by Osman et al. [50]. In addition, a high level of APEC resistance to medically important antibiotics, including β-lactams antibiotics, may have a high impact risk on humans [50]. In this study, only imipenem and polymyxin B demonstrated relatively lower resistance rates (1.7% and 0.4%, respectively). It might result from the lower number of clinical submissions in poultry.

Animal poultry and livestock are the most important reservoirs for pathogenic *E. coli* and the use of antibiotics is considered the most favorable factor in the emergence and the dissemination of antibiotics drug-resistance among animals and humans [51]. In the current study, 100% of the isolates were multi-drug resistant (MDR) compared to other findings reported from China (89.2%), South Korea (75%), and Brazil (71%) [50,51,52]. In addition, we found a similar MDR frequency between chicken (100%) and duck (100%) isolates. This result was different from the recent study in South Korea which reported 77.1% in chicken isolates and 65.5% in duck isolates [48]. The high MDR rates in our study demonstrate the high antimicrobial resistance and the inappropriate use of certain antibiotics in farm animals in China [53]. Therefore, there is growing evidence that APEC infections of animals and humans are becoming progressively difficult to treat in China [51].

In conclusion, a total of 230 APEC strains were isolated and characterized, which revealed a predominance of the O78 serotype as well as phylogenetic groups B2 and A. The APEC isolates in our study showed a high prevalence of virulence genes and significant antibiotic resistance rates. However, the APEC strains were not collected from all of China in this study. Although some relationships between virulence genes and serotypes, in the phylogenetic groups were observed, the mechanism of how these virulence genes transferred is not illustrated. Given the consistency between our data and those of previous studies, further studies are needed to assess the zoonotic potential of APEC as the reservoir of virulence genes and antibiotic resistance genes for other *E*. *coli* and bacteria.

## Data Availability

The data presented in the study are available in the manuscript.

## Supplementary Materials

The following supporting information can be downloaded at: https://www.mdpi.com/article/10.3390/vetsci9070319/s1, Table S1. Primers used for the detection of the virulence genes in APEC; Table S2. Serotypes of the 230 APEC isolates; Table S3. Phylogenetic grouping of the 230 APEC isolates; Table S4. Prevalences of virulence genes in the 230 APEC isolates; Table S5. Relationship between virulence genes distribution and serotypes; Table S6. Relationship between virulence genes distribution and phylogenetic groups; Table S7. Prevalence of antibiotic susceptibility testing; Table S8. Distribution of multi-drug resistant (MDR) APEC isolates; Table S9. Detailed information for the APEC isolates.
